# Mandated Treatment and Its Impact on Therapeutic Process and Outcome Factors

**DOI:** 10.3389/fpsyt.2019.00219

**Published:** 2019-04-12

**Authors:** Henning Hachtel, Tobias Vogel, Christian G. Huber

**Affiliations:** ^1^Forensic Department, Universitäre Psychiatrische Kliniken (UPK) Basel, Faculty of Medicine, University of Basel, Basel, Switzerland; ^2^Department of Adult Psychiatry, Universitäre Psychiatrische Kliniken (UPK) Basel, Faculty of Medicine, University of Basel, Basel, Switzerland

**Keywords:** perceived coercion, mandated treatment, stigmatization, therapeutic relationship, funtioning, recidivism

## Abstract

Court-mandated treatments imply a dual role for therapy providers not only of caring for, but also of having control over, involuntary clients. The impact of legal coercion on the therapeutic relationship and feelings of stigma is widely regarded as negative and detrimental for treatment outcomes. This point of view stands in contrast to advocates of the perspective that involuntary treatment can ameliorate social functioning and thus promote a better quality of life. Regarding other outcome measures, there is evidence that offender treatment is effective and leads to reduced recidivism in criminal behavior. This narrative review provides an overview of research assessing the effects of mandatory treatment on therapeutic process and outcome factors. We conclude that legal mandatory treatment does not have to necessarily result in perceived coercion and reduced satisfaction with treatment and that a caring and authoritative treatment style aids a favorable therapeutic alliance, motivation, and therapy outcomes.

## Introduction

Correctional treatment mandated by court is aimed at reducing recidivism in offending behavior. There is some evidence that this form of legal coercion can be effective in reducing the offending outcome (1), while other evidence suggests that mandated treatment is ineffective in reducing recidivism (2). The concept that legal coercion is inevitably related to perceived coercion with negative effects on treatment outcomes (3, 4) is widely held. In contrast, other evidence indicates that also voluntary clients can feel coerced into hospital admission (5) with resulting poorer satisfaction (6) and symptom change (7); legal detention had no association with perceived coercion (8, 9). These contradictory results hint at confounders and moderating variables influencing general treatment outcomes.

### Mandated Treatment and Therapy Facets

While mandated therapy provides external motivation to attend treatment, voluntary clients are normally believed to be intrinsically motivated. This view is challenged by studies that demonstrated perceived coercion in voluntarily admitted service users (10). Perceived coercion in voluntary samples had an unfavorable impact on long-term outcomes (11). While involuntary referrals by civil law and mandated treatment by penal law are a form of legal coercion resulting in external motivation, voluntarily admitted service users should at first glance be more motivated by their own innate psychological needs. The evidence that perceived coercion can also be present in voluntarily admitted clients resulted in statements that perhaps there are not so many differences between mandated and voluntary clients after all; a considerable percentage of clients are being coerced, whether it be legally or informally by family or employers, to be in therapy (12). This finding, however, has important implications for the stability of therapeutic interventions: behavioral changes last longer when they are the result of intrinsic motivation; extrinsically motivated behavior changes only last as long as extrinsic controls are in place (2, 13).

Literature suggests that mandated clients are more resistant to therapy than voluntary clients (14, 15). This may be a reaction to compulsory treatment as clients are more likely to resist their loss of freedom and independence (16, 17). A widespread model of motivation to change in generic psychiatry is the Transtheoretical Model (18), which suggests that recovery and the engagement with therapy is typically not a linear process but rather involves a cycling back and forth of the person’s perception of his or her problems and the level of behavior change. Therapy and engagement with the mandated client are further influenced by the forensic setting and need to be reflected in the therapeutic process (19). Frequent barriers confronted in mandated therapy are predominant male clients with more restrictive attitudes toward the changeability of psychiatric disorders (20), chronic course of severe mental illness, and high rate of comorbidity (21). Clients seem less inclined to seek help (22).

Within the scope of this narrative review, a literature search was conducted in PubMed, PsycInfo, and Google Scholar using various combinations of the following search terms: “mandated therapy,” “court ordered treatment,” “perceived coercion,” “therapy outcome,” “treatment outcome,” “therapeutic process factors,” “recidivism,” and “symptom levels.” From the resulting body of literature, domains were generated (stigmatization, functioning, therapeutic relationship, and satisfaction) and grouped according to clinical significance and empirical relevance for general therapy outcomes and factors. Papers were referenced in the review if they included relevant and additional insight into the respective domain; studies were not integrated or reproduced in their fundamental conclusions if publications with similar conclusions had already been discussed. The resulting summary of factors constitutes a compilation for service providers who are interested in the clinical application of the results. The quality of the therapeutic relationship is described as an important process factor in psychiatry and psychotherapy (23). The quality of the relationship between a service provider and a client is widely recognized as playing a key role in treatment adherence (24), symptom reduction, medication adherence (25, 26), outcome of psychotherapy and psychosis treatment (26–28), and quality of life (29). Positive effects of relationship quality were reported on client satisfaction, personal trust (30), and recidivism of criminal behavior. Literature suggests an association between lower dropout rates, better medication adherence, fewer readmissions, and improved symptom levels for clients suffering from schizophrenia spectrum disorders and the quality of the relationship between service provider and client (31–33). Coercive measures are believed to have a negative impact on the therapeutic relationship (34). A pronounced perceived coercion was reported to be associated with a poor rating of the therapeutic relationship (35). In forensic settings, these aspects gain even more impetus where the legal framework leads to more restrictive treatment requirements (23).

Experiencing stigmatization and accompanying discrimination are a powerful negative attribute in all social relations including psychiatric treatment. Feeling stigmatized has multiple negative consequences for mentally ill persons (36). Besides increased anxiety and stress, decreased functional outcome, loss of self-esteem and quality of life, and decreased social participation were reported (37, 38). As both mental health service utilization and treatment adherence are decreased through stigmatization, it can indirectly promote the aggravation of psychiatric symptoms (39, 40). Mandated clients have the extra burden of their dangerous or antisocial behavior. The existing—and quite limited—literature on stigmatization in forensic settings suggests that this would merit the inclusion of anti-stigma interventions in therapeutic programs (19).

According to literature of all available client-reported outcome measures, treatment satisfaction has the greatest evidence base (41). Improved satisfaction with services is associated with positive treatment outcomes including improved quality of life (42), higher levels of functioning (43), and reduced admissions (44). Compulsory actions and coercion were commonly described by clients as dehumanizing and detrimental to treatment satisfaction (45).

The limited systematic research on clients following coerced hospital admission and symptom change over time suggests some improvements (46, 47). As a limitation, clinical improvement has been commonly assessed on global functioning scales rather than validated symptom scales with usually small sample sizes (7). Recidivism in offending behavior is a peculiarity of mandated treatment and is normally not investigated in generic therapeutic research, while the influence of psychopathological symptoms on recidivism is of interest in forensic psychiatry.

So what does literature suggest about the impact of mandated therapy on the addressed therapeutic process and outcome factors?

## Stages of Engagement

While therapeutic stages in forensic settings are very similar to generic therapeutic processes, there are some peculiarities to consider. Mandated therapy can be divided into the initial task of stabilization and observation and the middle phase of remediation. The end phase includes rehearsal of skills, beginning detachment, and consolidation as preparation of leaving the scope for legal coercion (19). Important features of change for mandated clients include the realization of the need for therapy, coupled with a willingness to ask for and receive help (48). To counter the chronicity of a subgroup of clients who remain resolutely “unwilling” and therefore difficult to engage for many years, avoidance and needs resulting from complex life histories often influenced by trauma and experiences of abuse (49) are to be addressed. Frequently, cognitive behavioral therapy elements like motivational interviewing (50) are adapted in offender treatment as motivation for change cannot be assumed (51). The prolonged initial phase focuses on engagement, attendance to the therapeutic alliance, and the duration and intensity of treatment (19). Studies of readiness to change under legal coercion among adults with substance use problems provided mixed patterns of result but suggested a greater readiness to change after controlling for addiction severity, prior treatment history, and gender (3, 4). The prospective outpatient study (*n* = 295) was a heterogeneous, mixed gender sample of voluntary and legally coerced drug abusers with stages of change as an outcome measured by a self-administered instrument (4).

According to the Self-Determination Theory, factors that enhance versus undermine competence, autonomy, and relatedness yield enhanced self-motivation and mental health (52). Such an intervention style promotes a progress from amotivation, to passive compliance, to active personal commitment (52); these stages of motivation and engagement seem to be effective in general for a wide spectrum of service users irrespective of legal setting. In mandated treatment, only limited results hint at the generalizability of these results. However, favorable outcomes seem to result from interventions that encourage powerful attachment relationships, and communicate compassion, warmth, and intimacy (53), in short interventions, which promote a better therapeutic relationship.

## Therapeutic Relationship

Only sparse literature is available on the quality of the therapeutic relationship in mandated treatment. A cross-sectional study (*n* = 113) investigated adult male inpatients with a diagnosis of schizophrenia spectrum disorder being treated in general psychiatric wards and medium secure forensic psychiatric units *via* questionnaires (23): Self-referred clients reported a more positive therapeutic relationship than involuntary admitted clients in general psychiatry wards. Mandated treatment clients in forensic units gave an intermediate rating. There was no association with the clients’ legal status and the rating of the quality of therapeutic relationship or the service provider’s rating. Symptom severity and especially hostility were inversely related to the ratings of quality of the provider–client relationship. Generalizing these findings to females and service users without severe mental illness would be tentative, limiting the evidence base. However, the vast majority of patients of mandated treatment by penal law are male.

In the community, probationers and their officers believed that the quality of their relationship had an important influence on clinical and criminal outcomes (54). To better capture the dual role of providers of mandated therapy to care and to control, Skeem at al. (55) developed and validated a self-report-based questionnaire and probation officer form (the Dual-Role Relationship Inventory) for involuntary clients, which assesses the quality of the therapeutic relationship. It was found superior to a leading measure of therapist–client relationship quality in capturing the nature and effect of relationship quality in mandated treatment. The implication of this finding is that the dual role of mandated treatment is not adequately captured by “traditional” conceptualizations of the therapeutic alliance. More specifically, a “firm, but fair” approach with blending care and fairness with an authoritative (not authoritarian) style is more effective. This form of procedural justice (e.g., patients feel respected and experience a participatory decision making) was seen as crucial for experiencing less coercion even in involuntary settings (56–58). Probation violations and new arrests were predicted by the quality of synthetic relationship, e.g., the therapeutic approach of combining active listening and directive supervision without stressing a punitive orientation. This negative style of establishing control through authoritarian service provider confrontation within a session was labeled “Toughness.” This had deteriorating effects on relationships and future rule compliance mainly through client mistrust and treatment amotivation. The accompanying indifference to clients’ views and feelings, expectation of compliance, and punitiveness when expectations are not met seem to result in a negative struggle over issues of power and control. This rationale is in line with evidence that hospitalization, even when voluntary, was viewed as more coercive when clients rated their relationship with the admitting clinician negatively (35): The UK cross-sectional, mixed-gender study (*n* = 217) of consecutive admissions to acute adult wards measured perceived coercion, global functioning, and therapeutic relationship with self-reports. The examined disorders included affective and substance use disorders besides schizophrenia and others. Major limitations were the single-site design, the exclusion of a substantial proportion (about 22%) of possible participants because they were deemed too ill or too intellectually impaired, and retrospective measurements. A meta-analysis of the effectiveness of interventions of juvenile offenders (aged 12–21, outcome measure: recidivism, 548 independent study samples) in English-speaking countries emphasized the importance of a “therapeutic” intervention philosophy as opposed to a focus on deterrence, surveillance, or discipline (59).

In short, the interpersonal style influences the quality of the therapeutic relationship. The quality of the provider–client relationship shapes treatment outcomes more strongly than specific psychotherapy techniques applied (24, 60, 61). These findings seem to be present in juvenile and adult samples of voluntary and mandated treatment settings irrespective of gender and seem to be valid for different mental disorders. Evidence suggests that the quality of the therapeutic relationship is capable of being influenced in the same way in inpatient or outpatient settings; something not alike the feeling of stigmatization and devaluation.

## Stigma and Discrimination

There is a lack of research investigating the effect of mandated treatment on feelings of stigma. A Swiss study investigated the impact of different psychiatric service institutions on the stigmatization of mentally ill persons through a representative population survey (36): The desired social distance of the general representative population (*n* = 2,207) and therefore the stigmatization of mentally ill persons in this vignette-based study were lower in relation to psychiatric service use than to psychiatric symptoms; i.e., being treated in a psychiatric unit at a general hospital decreased stigmatization. It was of no relevance for stigmatization if hospitalization occurred in a general or forensic psychiatric clinic. Limiting the results is the fact that it is not clear how the attitudinal measure (i.e., the social distance scale) translates into real-world behavior.

Outpatient voluntary and court-ordered treatment for people (male and female adults) with serious mental illness were examined in the US study of Link and Castille (62). The observational study of a convenience sample (*n* = 184) was limited through a possible dropout bias and possible confounding variables. There was evidence supporting the hypothesis that self-reported feelings of coercion increased experience of stigma and devaluation. Further, there was a strong correlation between the number of involuntary hospitalizations and the current perception of stigma. This finding indicates that prior experiences with coercion influence subsequent experiences of coercion. Surprisingly, the ill-effects of perceived coercion and resulting lower self-esteem did not seem to affect illness-related social functioning or symptoms. This finding is in line with results from Ref. (10), which found no difference in functioning between involuntarily admitted clients and uncoerced voluntary clients (*n* = 169 mixed-gender inpatients with major mental illnesses) regarding longer-term therapy outcomes. Stated limitations include a moderate follow-up period (1 year after discharge) and completion rate of instruments, regarding engagement and functioning, which might have introduced a selection bias in these results.

In summary, what can be inferred from the sparse evidence is only tentative. Not the legal setting *per se* but rather perceived coercion seems to be linked to feelings of devaluation in adults. Also, having a history of coercive experiences makes it more probable to reexperience discrimination. As mentioned, lower self-esteem does not necessarily result in lower functioning (10), but is that true for treatment satisfaction as a major factor as well?

## Treatment Satisfaction and Functioning

An Irish multicenter study (*n* = 161 adult male and female participants with serious mental illnesses) observed a good overall level of satisfaction with services following voluntary and involuntary admissions (6). Experiences of physical coercion, low perception of being respectfully involved in a fair decision-making process regarding admission, and involuntary hospitalization were associated with lower service user satisfaction. In the same study, the therapeutic relationship was moderately correlated with the level of reported satisfaction with the service. Better global functioning and improved insight were associated with higher level of treatment satisfaction, emphasizing the relevance of service user satisfaction for general treatment outcome. A selection bias of uncompleted interviews and possible nonparticipants with higher probability of involuntary legal status were seen as main limitations.

The investigation of court-ordered outpatient treatment (*n* = 184 male and female adults with serious mental illness) in the United States observed improvements in symptoms, resulting in better social functioning and at a trend level better quality of life (62). This effect was countered and an erosion of quality of life was noted in study participants who self-reported elevated levels of coercion. Comparable long-term effects of voluntary and involuntary admissions to mental health services were reported regarding satisfaction and global functioning 1 year after discharge. This implied that clients feeling coerced to treatment can be subsequently engaged with a good therapeutic alliance (10).

In short, there is scarce information in adult service users with mental illness on global functioning and treatment satisfaction in involuntary settings. Literature could be interpreted to the effect that high levels of perceived coercion and low involvement of service users seem to be associated with lower treatment satisfaction and global functioning. Other outcome parameters like criminal recidivism and symptom change are the focus of more studies in forensic psychiatry.

## Symptom Levels and Criminal Recidivism

Symptom change (as an outcome factor for treatment effectiveness) after involuntary treatment in comparison to voluntary admission to services is often the primary focus of debate. A multinational European study investigating about 3,000 adult male and female inpatients of differing legal status and their subjective feeling of coercion concluded that, following coerced hospital admission, clients show, on average, moderate improved symptom levels after 1 and 3 months (7). This follow-up period could be seen as a limitation, as well as a possible selection bias through a high percentage of non-participants (about two-thirds). Besides higher baseline symptoms, legal voluntary status with feelings of coercion, and initial low treatment satisfaction, social factors like unemployment and living alone were important predictors for poorer symptom outcomes. Mandated treatment in the United States as evaluated in over 2,700 women with histories of abuse and co-occurring disorders equally demonstrated an improvement on psychiatric symptoms; the nationwide longitudinal study suggested that coercive status proved to be a significant main effect; i.e., being mandated was associated with greater improvement (63). However, women without drug abuse were not included and symptoms were evaluated by self-report.

The results on general and specific recidivism as a main treatment outcome in forensic therapy are partly inconsistent and reveal a great heterogeneity of results. While a meta-analysis (129 mostly US and Canadian studies, juvenile and adult samples of both gender, various treatment types and quality, no information on mental illnesses given but treatment targets specified) of mandated treatment of offenders was found to be ineffective on general recidivism, particularly in custodial settings, voluntary treatment was found to produce significant treatment effect sizes regardless of setting (2). However, mandated treatment was reported as effective on specific recidivism. It was reported that three decades of research into the effectiveness of legal coercion in the treatment of substance abusers have yielded inconsistent and inconclusive patterns (3). A more recent evaluation of specialized treatment on recidivism rates in Switzerland (*n* = 412 male adult offenders; mean follow-up, 7.9 years; mean duration of treatment in the intervention group, 4.5 years; overall about 85% psychiatric diagnoses of participants) revealed only a trend toward a positive treatment effect in violent and sexual offenders (64). The significantly higher criminal history of the intervention group and substantial missing information on diagnoses in the control group (i.e., the control group could have been less mentally burdened) constitute limitations of these results. A significant specific reduction in recidivism was, however, found by Lösel and Schmucker (65, 66) who, in two large meta-analyses (69 studies of adult and juvenile intervention and control group designs included in 2005; 29 study comparisons of males in group designs in 2015) on the effects of sexual offender treatment, reported a relative reduction in recidivism of 37% and 26.3%, respectively. The authors stated, though, that the evidence basis for sex offender treatment is not yet satisfactory and that there is a need of differentiated, high-quality evaluations. Results of a more recent meta-analysis (35 studies in 10 countries involving mostly violent offenders of heterogeneous samples and admission criteria) on general reoffending provided some evidence that clients discharged from forensic psychiatric services have lower offending outcomes than control groups discharged from prisons (1). A quantitative summary on meta-analyses from Andrews and Bonta (67) stated that rehabilitation programs of adult offenders that adhered to the Risk–Need–Responsivity (RNR) model have been shown to reduce recidivism up to 35%. This model proposes who should receive services in correctional settings (e.g., moderate- and higher-risk cases), treatment targets (criminogenic needs), and the most appropriate form of delivering therapy (e.g., cognitive social learning).

In juvenile offenders (aged 12–21, mostly male) a meta-analysis (548 mostly US study samples) stated a mean reduction in recidivism of 10–13% from a control group for interventions following a therapeutic and qualitative approach (59). The information on the interventions used was limited and target needs and types of recidivism were not differentiated. Worth noting in this regard is that literature reports aspects of residential youth care to be associated with repression and coercion (68).

In summary, literature suggests that criminal recidivism can be effectively reduced if treatment is evidence-based, supportive, and based on relational care (59, 69–72). These findings are replicated in juvenile and adult samples mostly with a male bias, but relatively independent of the treatment setting.

## Discussion

Evidence suggests that perceived coercion in treatment is linked to an impaired therapeutic process and outcome compared to voluntary treatment. While correlations have repeatedly been reported between perceived coercion and involuntary legal status (35, 73–75), some findings indicate that perceived coercion is not necessarily the result of mandated treatment status; i.e., feelings of coercion do not always follow a court order to therapy. Indicative of this consideration are findings that even physical coercive measures are shown to be a separate entity from procedural justice and perceived pressure (76). Further, the therapeutic relationship seems to confound legal status as a predictor of perceived coercion (35). The feeling of coercion seems to be dependent of various determinants, many of which depend on the quality of the relationship with the service provider, and clinicians should therefore routinely consider that involuntary and voluntary clients have the potential to experience interventions as coercive (45). This is in line with study results comparing reports of coercion in clients mandated to outpatient treatment and a control group that reported no significant differences (62). In qualitative analysis regarding perception of coercion, three themes were identified to be linked to feelings of coercion: viewing the service institution as ineffective and other treatments as more appropriate, not participating in the admission and therapy, and the missing feeling of respect (5). These themes point consistently to low procedural justice as being important in the development of experiences of coercion.

Discrimination does not seem especially associated with a forensic setting (36), but rather perceived coercion is linked to feelings of stigma; furthermore, a history of coercive experiences facilitated the reexperience of devaluation (62). Similarly, high levels of perceived coercion and inability to involve service users seem to result in lower treatment satisfaction and global functioning (6, 10).

Ethical considerations place a great emphasis on personal autonomy and self-determination of clients (77). Regarding mandated and forced therapy, there is subsequently a strong focus on what circumstances can justify infringing these values. The argument being made for coercive treatment is that in certain circumstances, i.e., when certain mental disorders determine the behavior of the client and therefore constitute a form of coercion themselves and result in an inability to consent, it can be justified to perform compulsory treatment to restore the capacity for autonomy (77). Other arguments point out that perpetuating the assumption that all types of leverage including mandated therapy amount to coercion is misleading and unhelpful (78). The lines between bargains and coercion are not easily drawn, and under certain conditions, several forms of mandated treatment are better understood as the product of negotiation and voluntary agreement (e.g., access to housing, avoidance of jail) (79). Similarly to the ethical importance of self-determination, therapeutic considerations stress three innate psychological needs: a secure relational base (relatedness), the feeling of volition (autonomy), and the feeling of being efficacious with respect to activities (competence) (52). The fulfilment of these needs seems to predict mental well-being and facilitate the integration of extrinsic motivation.

Research on the therapeutic process and outcome factors described above suggests that mandated treatment can also be associated with results comparable to and, in some cases, better than voluntary treatment. While perceived coercion, resistance, and lack of intrinsic motivation to change are more likely to be present at the beginning of treatment, these do not seem to be determinative of the mentioned therapeutic factors and outcomes as therapy progresses (12). These treatment barriers appear to be accessible to a specific therapeutic relationship quality and interventions particular in mandated therapy settings. According to the stages of therapy engagement, a caring, fair, and trust-evoking quality of therapeutic interventions blended with a firm but not authoritarian or punitive control seems to be necessary to change unwillingness or amotivation to therapy engagement and the will to change (34, 55). Paired with the effort for procedural justice as far as legal constraints of mandated therapy allow, the respectful involvement into treatment might assuage possible experiences of coercion, resulting in a better quality of therapeutic relationship, more treatment satisfaction, and less experience of stigmatization. A possible distinct advantage of mandated therapy is the (at first more extrinsically motivated) consistent and longer attendance in treatment than in voluntary settings. This circumstance could lead to a more intrinsic motivation in offender clients along the therapeutic process (12). This factor and the additional provision of supportive aftercare may explain good levels of satisfaction in involuntary treatments along the way and resulting better levels of functioning (1, 6).

There is some evidence that mandatory treatment does achieve the required treatment targets of legal coercion: reduction of recidivism and symptoms. Reports of high symptom loads of clients in involuntary and forensic settings (7, 21) seem to moderate low improvements of symptoms (7). The heterogeneity of evidence on the reduction of reoffending might be attributable to different admission criteria of institutions, varying treatment principles and quality, variability in sample compositions, missing information on client location at discharge, and varying quality of included studies in meta-analyses (1). While the odds of mandated therapy appear to be stacked against favorable outcomes at the onset, there is evidence that mandated treatment can work in a wide range of specific criminal behavior like violent or sexual offending (2, 12). Adhering to Risk–Need–Responsivity principles in forensic therapeutic settings has repeatedly generated lower recidivism rates with a substantial effect [i.e., mean effect sizes (*r*) of up to 0.29] (67). In institutional youth care, effects of coercion might be associated with residential care (72). However, evidence from juvenile offender samples suggests that effective treatment is not highly context dependent; i.e., the intervention effects are even robust in institutional environments with more potential adverse conditions (59).

The nature of the search and inclusion process employed in the current narrative review limit the generalizability of some of the reported results. The evidence base of mandated therapy is small regarding various facets of therapy process factors. To complicate matters further, mandated therapy can occur in different forms of legal coercion (i.e., by civil or penal law). Therefore, the current review provides a clinical overview to summarize the most relevant results for service providers and aims to raise awareness of important issues associated with mandated therapy for future research. Another major limitation of the current review is that the present overview—due to the state of the literature—cannot make specific statements on various determinants of mandated treatment outcome (i.e., depending on inpatient or outpatient setting, male vs. female samples, sex offenders/non-sex offenders, and heterogeneous types of mental illnesses). In addition, most of the reported studies were observational, cross-sectional studies, which could only report associations and no causalities.

In conclusion, treatment outcomes in different domains seem to be linked to the client’s motivation to attend treatment and the feeling of being coerced into therapy, regardless of mandate (2). It has been argued that there is, potentially, an element of coercion in every clinical encounter (80) and the perception of coercion has a variety of determinants, many of which are dependent on the quality of relationship with the service provider (45). Therefore, reducing feelings of coercion might improve treatment outcomes, prevent disengagement from services, and ameliorate therapeutic relationships (5). Facilitating the integration of extrinsic motivation through participatory decision making and interpersonal contexts of relatedness and security produces maintained change (52). Service providers should therefore be encouraged to find the right balance between control and flexibility (70): A dual-role relationship (“firm but fair”) can help to motivate offenders to engage and stay in therapy (55) and reduce offending behavior (34, 35), despite lack of motivation and possible high symptom load. In this regard, the more consistent and longer attendance due to legal framework with provision of supportive aftercare (70) can enable motivational interventions and strengthen therapeutic relationships.

## Author Contributions

HH and TV wrote the initial article, and CH revised the paper critically for important intellectual content.

## Conflict of Interest Statement

The authors declare that the research was conducted in the absence of any commercial or financial relationships that could be construed as a potential conflict of interest.
